# The Hygiene of Motherhood

**Published:** 1890-12

**Authors:** John Sheppman


### THE HYGIENE OF MOTHERHOOD.

BY DR. JOHN SHEPPMAN.

PART SECOND.

We will continue our investigations upon diet and the immediate influence which it exerts upon the health of all people. Presuming that my last article embraced the prominent objections to be found in animal foods, I shall now endeavor to define some of the other characters with which we are provided for daily consumption. There is nothing within the range of human edibles that is more universally used and desired than bread. Indeed, there is no nation in the world that does not have some form of this particular food. In Biblical history we are informed that the angels were fed and feasted upon unleavened bread, and that Pharaoh was instrumental in the execution of the chief baker upon the interpretation of Joseph. We have conclusive evidence to believe that baking was practiced as far back as the stone period, and that very stringent laws were enacted respecting the size and quality of the loaf, and to prevent unscrupulous men from swindling, or imposing upon the rights of their generous patrons.

In the fall of 1265, the English Parliament passed an act by which the price of bread was brought to a marked or standard uniformity ; adulteration was made an offense, punishable by a fine and by prison, and very often by a severe and prolonged confinement in the ignominious pillory. The chief prohibited article used by the transgressor seems to have been alum. With its addition flour of an inferior grade can be made to assume a perfect whiteness, and other ingredients which lack the nutritive qualities of wheat can be successfully substituted.

How much adulteration is carried on at this time cannot be definitely stated ; for, when we consider the mechanical improvements of the miller, and the modern ingenious methods of kneading, we can hardly commit ourselves to believe that it would be profitable for any one to make bread that is not pure, wholesome and entirely free from all chemical and foreign substances. Many of the baking powders that arewidely known and advertised as "pure" contain alum, and its presence is regarded by high medical authorities to be a direct infringement upon the general laws of health. Alcoholic yeast is preferable for raising dough from a sanitary view : Not only is the bread made with it lighter, but through the process of vaporizing the vesiculations become more diffused, and the action or change of starch into glucose is a particular digestible advantage in its favor.

It was formerly customary to use the entire wheat berry in the preparation of dough ; this, of course, made the bread considerably darker, but allowed it to contain the principal nutritive properties with which nature has bountifully endowed it. The size of the loaf should not be too large, as the amount of heat required to thoroughly bake the inner portion would have to be so intense as to cause the outside crust to burn before it accomplished its centripetal purpose. In mixing flour it is best to use soft water, as the bread made in this way is considerably better, and remains fresh for a longer period. It is an excellent plan to place a cup of water, mixed with half a teaspoon of salt, in the oven while your bread is baking. This will insure softer bread, and with a lighter crust.

Pastry, and in fact all carbonaceous foods, are more or less injurious, and may be looked upon as the principal conductors to biliousness, and to many other serious and chronic derangements of the liver. An excess of sugar is particularly harmful, and tends to produce fatty degeneration of the liver and kidneys, and without doubt takes an important part in the promotion of Bright's disease. The decaying influence which it has upon the teeth is already very well known; and some physicians have gone so far as to declare that cataracts of the eyes can be produced by its inordinate use. All sugar, and sugary articles can easily be dispensed with, for the amount required by the human system will always be found in the starchy substances which are converted by the action of the liver into glucose in the general process of digestion. It would not be a very difficult matter for many of our young ladies to master their desire for sweetmeats if they only knew how much their beauty hangs upon the quality and quantity of the food they eat, and that the face, with its many charms, is soon blighted with a manifestation of the painful condition of the inner organs, which are constantly straining their efforts to perform their natural functions. Too much, and too little food, are the two extremes which are equally detrimental, and the individual should learn to eat with judgment, remembering the folly of sacrificing health and comfort for the mere gratification of the palate.

> Health must be there, or beauty cannot be :
> The sunken, languid eye, the pallid cheek,
> The lax and purple lip, but move the mind
> To pity—not to love.

In direct opposition to sugar we have salt. This article must not be condemned, for it appears in nearly every portion of the human body. It contains the elements which are required for the growth of the teeth, hair and nails ; it aids digestion, stimulates the appetite, and no doubt renders valuable assistance in preventing alimentary fermentation. With salt, as with every thing else, we must not use abnormal amounts. When taken in excess it irritates the throat, the stomach and the tissues, and probably conduces to a severe cancerous state of the liver.

Eggs are generally considered healthy when properly prepared by the cook. Whether it is worth an act of legislation to classify them, as they do in London, is very hard to tell. There they have "new laid eggs," or "first rate eggs," "fresh eggs," or "moderately good eggs, and "eggs." It is very positive that "eggs" of the third class cannot be too far gone, for nature has generously adapted the unmistakable olfactory method which will cause even the most hungry mortal to retire from their obnoxious presence. The best way to cook eggs is to bring water to the boiling point, drop in as many as you wish, remove the pot from the fire and allow it to stand for five minutes. When the eggs are removed they will be found to be thoroughly cooked, and in a very digestible manner.

Vegetables of all available kinds should always be found upon our tables, as they have a direct chemical and remedial action upon our bodies. Onions and asparagus are particularly recommended for rheumatism and rheumatic gout ; cauliflower has a tonic effect upon the system ; and boiled peas contain ninety-three per cent. of nutriment,which is almost equal to the value of wheat bread. Potatoes are certainly a very desirable part of our meals, and lettuces, carrots and cabbages would be much easier digested but for the artificial methods of cooking, and seasoning which are employed by the modern civilized cook. Vegetables should never be put into the cellar, as many of them contain acids which will absorb the poison of the ground air, and, if eaten, will prove very unhealthy; and if allowed to remain, will rapidly decompose and fill the air, which arises to the upper rooms, with a poison that will undoubtedly cause much mischief. Potatoes should not be exposed to the sun, but kept in some dry place where the light and air can always strike them.

The extent to which adulteration of milk is carried on in these enlightened times is certainly very appalling, and proves that "man's inhumanity to man" is increasing, instead of decreasing. The first among the guilty ones in this respect is the owner of the cows—the farmer, or the dairyman. Upon him depends the first quality of the milk and whether it is taken from diseased cows, or from those who are well fed, and in a fit and perfectly healthy condition. It may seem strange to some of my readers to learn that if a cow is fed upon green corn, or any thing that will have a purging effect upon it, the same affliction will visit the children who will drink of the milk which was taken from the cow at that particular time. By this point we will see the necessity of educating the farmers on the proper care, and the food which should be given to their cows in order to secure perfectly pure milk. Many of the Germans are accustomed to boil their milk as soon as they receive it in the morning. This is an excellent example of hygienic prudence which would be well for all our women to follow. There are few articles which are more susceptible to disease germs than milk, and the practice of sterilizing it is one which should be heartily commended.

The enormity of the crime of adulteration should be more widely known, and the court respecting it should regard the offender with as little mercy as he has shown towards the hundreds of murdered ones who have sacrificed their lives upon the altar of impure milk. Over one-third of all the children born in this world die before their fifth year, and nearly all these deaths can be traced to an impurity in the milk supply, which man, with his wild and thoughtless grasp for wealth, will stain and corrupt himself to introduce. The time is not far off when all intelligent people will be brought to a realization of the horrible existing facts, and when every one will urge upon their representatives, and upon their men of influence, to make one gigantic effort for the suppression of this murderous evil.

Who has not gazed with admiration upon the innocent pranks of childhood? and who has not been charmed by the rosy smiles and the bright sparkling eyes of the healthy infant? What a contrast! to turn from this picture and to face the one who, meekly and sad, lies in silent agony, mercilessly permitted to suffer, until, by the soothing impulse of death, she—"like a lily drooping, bows her head and dies."

---

